# Authors’ Reply: Comment on “An Online Intervention Comparing a Very Low-Carbohydrate Ketogenic Diet and Lifestyle Recommendations Versus a Plate Method Diet in Overweight Individuals With Type 2 Diabetes: A Randomized Controlled Trial”

**DOI:** 10.2196/jmir.8776

**Published:** 2018-05-01

**Authors:** Laura Saslow, Ashley E Mason, Sarah Kim, Veronica Goldman, Robert Ploutz-Snyder, Hovig Bayandorian, Jennifer Daubenmier, Frederick M Hecht, Judith T Moskowitz

**Affiliations:** ^1^ School of Nursing Department of Health Behavior and Biological Sciences University of Michigan Ann Arbor, MI United States; ^2^ University of California, San Francisco San Francisco, CA United States; ^3^ University of Michigan Ann Arbor, MI United States; ^4^ University of California Berkeley Berkeley, CA United States; ^5^ Institute of Holistic Health, Department of Health Education San Francisco State University San Francisco, CA United States; ^6^ Feinberg School of Medicine Northwestern University Chicago, IL United States

**Keywords:** low-carbohydrate diets, type 2 diabetes

This letter is in response to the letter from Dr Andrew Reynolds [1] about our publication, “An Online Intervention Comparing a Very Low-Carbohydrate Ketogenic Diet and Lifestyle Recommendations Versus a Plate Method Diet in Overweight Individuals With Type 2 Diabetes: A Randomized Controlled Trial” [2].

As Dr Reynolds notes, our study had several differences between the two groups including different diets, psychological tools, sleep education, and number of lessons.  Given the combination of intervention components, we did not attribute all of the differences in outcomes to the nutritional composition of the diet, but to the diet “and lifestyle recommendations.” It was our goal, if this preliminary trial showed promise, to follow up with factorial screening experiments to better assess each component of our multicomponent very low-carbohydrate diet intervention. We are currently testing our very low-carbohydrate diet intervention in this way, and we hope to vary the dietary component in future research. We agree that a randomized trial that varies only diet is an ideal way to ascertain to what extent the nutritional composition of the diet, specifically, contributes to the outcomes.

However, important information can be learned about the overall effects of diet and lifestyle interventions without such screening experiments and intervention optimization. For example, the landmark Diabetes Prevention Program trial, with more than 3000 patients with prediabetes, compared a multicomponent low-fat, calorie-reduced diet and lifestyle intervention to metformin or medicine placebo groups [3]. The intact, multicomponent program is now used nationally, but to our knowledge, no research has carefully varied the dietary component to examine if the low-fat dietary recommendations are optimal. Despite this, this multicomponent program is supported by the Centers for Disease Control and Prevention and will be soon reimbursed by Medicare. Even so, we believe that the field needs to do more screening experiments and intervention optimization.

We acknowledge the baseline differences on some of our outcome measures. This can happen even with classic randomized controlled trials. Nevertheless, our statistical evaluations emphasize interaction effects and, as such, they emphasize relative changes and fully incorporate baseline information. Therefore, we disagree that the interaction effects are due primarily to these baseline differences. With respect to a post-hoc power analysis, a power analysis reveals the *likelihood* of observing a significant effect. We disagree that once a study has been completed there is any value to a post-hoc power analysis. After study completion, we know for certain whether effects are significant (“power” = 100% for significant effects, and 0% for non-significant effects), and these results utilize observed variability rather than estimates of variability that would have informed a pre-study analysis.

We agree that meta-analyses are ideal for judging the effects of different diets, however, we have a different interpretation of the meta-analysis to which he refers [4]. This meta-analysis of carbohydrate-restricted trials in type 2 diabetes itself notes that the “effect on glycemic control increased with the reported degree of carbohydrate restriction,” and that “recent trials suggest that LCD [low-carbohydrate diets] may be superior to HCD [high-carbohydrate diets] with respect to glucose level and postprandial excursions, but only as long as the subject adheres to the diet.” This suggests that when individuals can maintain adherence to long-term carbohydrate restriction they experience improved glycemic control. Moreover, only two of the seven studies in this meta-analysis followed participants beyond 12 months. We view this meta-analysis as support for the short-term benefits of low-carbohydrate diets in the treatment of type 2 diabetes, and that it highlights a need to conduct longer trials of low-carbohydrate diets that focus on long-term dietary adherence.

We appreciate the opportunity to respond to Dr Reynolds’ letter.
